# Cases of Idiopathic Glossitis

**Published:** 1831

**Authors:** 


CASES OF IDIOPATHIC GLOSSITIS. By MR. ORGILL.

CASES of this kind are certainly not common, and when they occur they deserve notice. Mr. Orgill has briefly published the particulars of three in our Glasgow contemporary for February of the present year.

*Case* 1. A farmer, aged 50, complained of much difficulty of deglutition, for which some common remedies were prescribed, and a week afterwards presented himself to Mr. Orgill. The left half of the tongue was so much swollen as to prevent articulation and deglutition; the right half was natural; the pulse was natural. Six ounces of blood were abstracted by leeches and a cupping-glass, applied to the root of the tongue externally, but this gave little relief, and Mr. Orgill introduced a scalpel flat on the dorsum of the tongue, and made two incisions half an inch deep, from as far as the instrument reached to the tip. The incisions bled pretty freely, and the swelling was a good deal reduced, but in the evening it was become as great as ever. It was scarified still more deeply, and a castor-oil enema prescribed. This also gave great relief for a season, but next morning the swelling had returned, with a peculiar lividity at the tip of the diseased half of the organ. An incision an inch deep was made with a scalpel, a gush of most offensive pus ensued, and in eight days the tongue was well. The sensibility on the affected side continued impaired for a year afterwards, but then it was gradually restored.

*Case* 2. A sailor, æt. 35, after languor and some rigor, complained of difficulty of deglutition, and next day the left half of the tongue was swollen to three times its natural bulk, and very painful. Its surface was foul, except at tip, which was very clean and red; the median line abruptly bounded the enlargement. There was much difficulty of articulation and deglutition, with fever. *V.S. ad* ℥xx.—*brisk purgative.* Next day the tumefaction and pain seemed again to be on the increase. *Five leeches to the tongue—cathartic repeated.* The disease rapidly abated, and on the fourth day the organ was perfectly healthy.

*Case* 3. A woman applied to Mr. Orgill with inflammation of the whole tongue, terminating in suppuration of the right half. She was relieved by scarifications, discharge of the pus, and cathartics. Some months afterwards she was attacked with the same complaint, and there was here a peculiar lividity and smoothness at the tip, on the side which suppurated.

---

